# Chronic pain after orthognathic surgery: a retrospective single-center study

**DOI:** 10.22514/jofph.2026.042

**Published:** 2026-05-12

**Authors:** Sakari Kettunen, Olli-Pekka Lappalainen, Laura Nykänen, Aleksi Haapanen, Antti Asikainen, Jussi Furuholm, Johanna Snäll

**Affiliations:** ^1^Department of Oral and Maxillofacial Diseases, University of Helsinki and Helsinki University Hospital, P.O. Box 320, 00029 Helsinki, Finland

**Keywords:** Orthognathic surgery, Chronic postsurgical pain, Trigeminal nerve injury, Psychiatric aspects, Postoperative period, Retrospective studies

## Abstract

**Background**: We aimed to clarify the occurrence and variables associated with postoperative chronic postsurgical pain in orthognathic surgery patients (OS). **Methods**: This retrospective single-center study included patients ≥18 years old undergoing bilateral sagittal split osteotomy (BSSO) with or without Le Fort I osteotomy between January 2016 and December 2022. The outcome variable was the occurrence of chronic postsurgical pain three months after OS. SPSS software (IBM Corporation, 28.0.0.0) was used to analyze the associations between predictor variables and outcome. **Results**: Chronic postsurgical pain was observed in 7.9% of the 317 patients included in this study. In univariate analysis, the outcome was predicted by older age (odds ratio (OR) = 1.044; 95% confidence interval (CI): 1.003–1.087; *p* = 0.033) and use of early gabapentinoid medication at hospital discharge (OR = 3.526; 95% CI: 1.286–9.666; *p* = 0.014). In multivariate analysis, only early gabapentinoid medication predicted outcome independently (adjusted odds ratio (aOR) = 2.975; 95% CI: 1.055–8.388; *p* = 0.039). **Conclusions**: Chronic postsurgical pain represents a significant postoperative disadvantage in OS, yet surgical factors appear to have limited influence on its development. More detailed information on other variables, such as psychosocial factors and resilience, is needed to predict postoperative pain in this specific patient group.

## 1. Introduction

The risk for inferior alveolar nerve (IAN) damage in oral and maxillofacial surgery is proposed to be the highest in orthognathic surgery (OS) [1], and chronic neuropathic pain is a major risk in OS [2, 3, 4]. Considering the elective nature of the surgery, OS professionals should be able to identify protective and exacerbating factors before surgery that could forecast the course of developing postoperative neuropathic pain. Pain prevention should be a target at every stage of surgical treatment.

Chronic postsurgical pain (CPSP) is defined in the International Classification of Diseases 11th edition (ICD-11), developed through collaboration between the World Health Organization (WHO) and the International Association for the Study of Pain (IASP), as pain developing after a surgical procedure or a tissue injury that persists or recurs for at least three months after surgery, excluding other causes such as malignancy, infection or pre-existing pain [5, 6, 7]. CPSP may frequently have a neuropathic component, signifying that the pain is caused by a lesion or a disease of the somatosensory system [5]. A key feature of neuropathic pain is the combination of sensory loss with paresthesia, hyperesthesia, or allodynia in a neuroanatomically logical site [5]. Neuropathic pain tends to be more chronic than nociceptive pain [8] and neuropathic pain is common in iatrogenic nerve injuries [9, 10]. Neuropathic pain occurring after OS is mostly recognized as posttraumatic trigeminal neuropathy (PTTN) and is a result of iatrogenic trigeminal nerve damage during surgery. Persistent scorching and/or shooting pain with a clear history of trauma is one of the primary features of PTTN [11, 12]. Recently, PTTN has been reported to be poorly recognized by dentists in oral healthcare [13].

Iatrogenic nerve injury to the IAN can be caused by laceration [14], exposure [14], compression [15, 16], stretching [17, 18], choice of technique [19], or incorrect use of surgical equipment. These injuries can lead to neurosensory disturbances in the region innervated by the nerve [14, 15, 17, 18, 19], and the occurrence of neuropathic pain seems to be associated with axonal injuries [20]. Symptoms of neurosensory disturbance can be present even if no injury is macroscopically visible [14], and studies have shown that sensory disturbances can be observed even before splitting of the mandible in OS [21]. Sensory loss is common after OS, and it seems to be highest at one month, decreasing during a one-year follow-up [17, 22, 23]. Most patients show significant recovery from sensory loss by two years after surgery [24]. Age has been identified as a factor associated with prolonged recovery after IAN injury [17, 21, 24, 25].

Failure to treat postoperative pain can lead to a cycle of physical and psychological problems [26]. In surgical settings, not specifically in OS, preoperative anxiety [27, 28, 29, 30], pain catastrophizing [28, 29, 31, 32, 33], psychological vulnerability and distress [8, 34, 35], and depression [27, 29, 34, 35, 36] have been associated with acute and chronic postoperative pain outcomes. Is this association present in OS patients? OS patients [37, 38] and patients with orofacial pain [4] have been reported to have psychiatric morbidity pre- and postoperatively, suggesting that OS professionals may be able to predict postoperative pain outcomes through psychiatric anamnesis conducted before surgery.

Aims of this study were to find patient- and surgery-related variables associated with chronic postsurgical pain and to investigate the occurrence of chronic pain in OS. We hypothesized that patients with chronic postsurgical pain in OS could be predicted by identifying patient- and surgery-related variables.

## 2. Materials and methods

### 2.1 Study design

A retrospective, single-center study of patients treated with OS was conducted at the Department of Oral and Maxillofacial Diseases, Head and Neck Center, Helsinki University Hospital, Helsinki, Finland. All surgeries were conducted by both senior consultants and surgeons in specialized training under supervision, ensuring adherence to standardized surgical techniques. The hospital database was manually reviewed for the electronic medical records of all patients who underwent OS between 01 January 2016 and 31 December 2022.

### 2.2 Inclusion and exclusion criteria

This study included patients aged ≥18 years who underwent bilateral sagittal split osteotomy (BSSO) without or with Le Fort I osteotomy (bimaxillary osteotomy), with ≥6 months of postoperative follow-up. Patients with developmental or intellectual disabilities, oral cancer, or secondary surgery due to a previous facial surgery or fracture were excluded. Additionally, patients with the presence of chronic pain conditions (active pain treatment with opioids, gabapentinoids, and/or amitriptyline) before surgery or those who received reoperation ≤3 months after primary surgery were excluded.

### 2.3 Study variables

The outcome variable was the occurrence of chronic postsurgical regional pain after OS in the operation area, as newly developed pain requiring medicinal intervention and persisting for at least 3 months after surgery. Data were included if the pain required medical intervention evaluated by the maxillofacial surgeon with pain medication other than non-steroidal anti-inflammatory drugs, paracetamol, or mild opioids commonly used during the acute postoperative healing period (pregabalin, gabapentin, amitriptyline, nortriptyline, duloxetine, and/or carbamazepine). Pharmacological treatment was prescribed according to the standard postoperative analgesic protocol of our institution. The typical starting dose was 150 mg of pregabalin divided twice daily, and adjustments were made by increasing titration and/or combining with previously mentioned medication based on pain intensity, sedation or comorbidities. Data for the prevalence of chronic pain (pain requiring treatment with medication) at 6 months and 12 months after surgery were also collected.

Predictors comprised patient- and surgery-related variables. Patient-related variables included age, sex (male/female), body mass index (BMI), history of alcohol and/or substance abuse, combined oral contraceptive medication, and preceding mood and/or neurotic, stress-related, and somatoform disorder (International Classification of Diseases 11th edition (ICD-11), mental and behavioral disorders groups F30–49, excluding bruxism F45.8.) [7]. Alcohol and/or drug abuse history was determined according to the Finnish Current Care Guidelines [39]. Surgery-related variables comprised surgical procedures classified as BSSO or bimaxillary surgery, perioperative dexamethasone administration grouped as ≤10 mg or no dexamethasone or >10 mg of dexamethasone, degree of manipulation of the IAN during surgery (grouped as IAN exposed, IAN dissected from the underlying bone or other nerve proximity surgical adjustment, laceration or loss of continuity of IAN or the accessory nerve), degree of mandibular transfer (grouped as advancement or setback), and type of osteosynthesis (custom/standard plates or combination of the two). Data were also collected on early gabapentinoid use (gabapentin, pregabalin) when these medications were prescribed by the maxillofacial surgeon or recommended by the anesthesiologist during postoperative discharge. Only patients without preceding chronic or neuropathic pain were included in this assessment. This variable represents the patient’s medication status at the time of hospital discharge, so it is treated as a discharge-level indicator rather than a preoperative risk variable in the analysis. Patients receiving gabapentinoids at discharge from the hospital were considered positive for the outcome (chronic pain at 3 months after surgery) if, at three-month follow-up, they continued to experience pain that required ongoing or increased pharmacological treatment.

## 3. Statistical analysis

SPSS software (version 28.0.0.0, IBM Corporation, Armonk, NY, USA) was used for the statistical analysis. Differences between patients grouped by categorical variables were evaluated using Pearson’s Chi-squared test. Means, minimums, maximums, and medians were calculated for the applicable variables. Logistic regression analysis was used to analyze the relationship between the variables. Given the limited number of events (n = 25), including all predictors would over-parameterise the multivariable model. To avoid instability, only variables with *p* < 0.05 in univariate analyses (age and early gabapentinoid use) were included in the model. Thus, the multivariable model reflects discharge-stage risk indicators rather than preoperative predictive factors. A significance level of *p* = 0.05 was selected for the analysis. Fig. 1 was created with Adobe Photoshop (version 26.11.0, Adobe Inc, San Jose, CA, USA) and Microsoft Excel (version 16.76, Microsoft Corporation, Redmond, WA, USA), Fig. 2 was created with R software (version 4.4.2, R Foundation for Statistical Computing, Vienna, Austria).

**Fig. 1.** An overview of patients with chronic pain at different stages during postoperative follow-up. Each column demonstrates the number of patients with chronic postsurgical pain at 3, 6, and 12 months after surgery and the comparison of mean and median ages between the groups.

**Fig. 2.** The distribution of patients and their ages with postsurgical chronic pain. The distribution is demonstrated by each dot representing a patient with chronic postsurgical pain, with mean age segments at 3, 6, and 12 months after surgery.

## 4. Results

The study population included 340 patients, and 317 patients (37.5% men and 62.5% women) were included in the final analyses after applying inclusion and exclusion criteria. Patients’ perioperative age ranged from 19 to 58 years (mean 33.8, median 31.2) (Table 1). BSSO exclusively was a more common surgery type (55.5%) than bimaxillary surgery (44.5%), and custom plates alone were the most common type of osteosynthesis (49.2%), followed by standard plates (46.4%) and a combination of the two (4.4%).

**Table 1.** Descriptive statistics of 317 patients undergoing orthognathic surgery.

Characteristics		No. of patients	% of 317
All		317	
Sex			
	Male	119	37.5
	Female	198	62.5
Age, yr			
	Mean	33.8	
	Median	31.2	
	Range	19–58	
Body mass index			
	Mean	24	
	Median	23.4	
	Range	16.3–34.4	
Alcohol and/or drug abuse			
	Yes	11	3.5
	No	306	96.5
Combined oral contraceptive medication, women			
	Yes	35	17.7
	No	163	82.3
Preceding mood disorder or neurotic, stress-related, and somatoform disorder			
	Yes	52	16.4
	No	265	83.6
	- Mood disorder	27	8.5
	- Neurotic, stress-related, and somatoform disorder	15	4.7
	- Both	10	3.2
Regular gabapentinoid medication after discharge from hospital			
	Yes	30	9.5
	No	287	90.5
Dexamethasone			
	Low (≤10 mg)	200	63.1
	High (>10 mg)	117	36.9
Surgery type			
	Bilateral sagittal split osteotomy	176	55.5
	Bimaxillary osteotomy	141	44.5
Manipulation of inferior alveolar nerve (IAN)			
	Yes	233	73.5
	No	84	26.5
	- IAN exposed	170	53.6
	- IAN dissected from underlying bone or other nerve proximity surgical adjustment	50	15.8
	- Laceration or loss of continuity of IAN or accessory nerve	13	4.1
Osteosynthesis type			
	Standard plate	147	46.4
	Custom plate	156	49.2
	Combined	14	4.4
Mandibular transfer			
	Advancement	286	90.2
	Setback	31	9.8

The incidence of chronic postsurgical pain is presented in Fig. 1. A total of 25 patients (7.9%) had chronic pain after OS evaluated at 3 months postoperatively. Most of these chronic pain conditions were assessed to be of neuropathic origin by fulfilling the diagnostic criteria; one patient had severe chronic temporomandibular joint-related disorder requiring pregabalin medication for treatment of pain. At the 6-month follow-up, 5.7% still had pain requiring treatment, and the corresponding prevalence at 12 months was 1.6%. No new chronic pain conditions were reported during follow-up, and the prevalence represents the same individuals who were treated for chronic pain at 3 months after surgery.

The distribution of patients and their ages with mean segments at 3, 6, and 12 months after surgery is presented in Fig. 2. Patients with chronic pain at 12 months after surgery had higher mean and median ages than patients at 6 months after surgery. A similar trend is demonstrated with patients at 6 months having higher mean and median ages than patients at 3 months after surgery.

Associations between chronic pain and age are presented in Table 2. Patients without chronic pain were younger (mean 33 years, median 30 years) than patients with chronic pain (mean 37.4 years, median 36 years) (*p* = 0.030), and when comparing groups of under 30 years and 30 years or older, chronic pain was more common in the older group (*p* = 0.032, risk ratio (RR) = 2.536).

**Table 2.** Associations between age and chronic pain in patients undergoing orthognathic surgery and calculation of risk ratio for complications between age groups.

		Patients with chronic pain		Patients without chronic pain		*p*	Effect size if significant	RR	95% CI
		n	%	n	%				
Age group									
	<30 yr	6	4	135	96	**0.032**	0.121	2.536	1.041–6.182
	≥30 yr	19	11	157	89				
Age, yr									
	Mean	37.4		33.0		**0.030**	0.454		
	Median	36		30					
	Range	23–58		19–57					

*RR: risk ratio; CI: confidence interval. The p-value is bolded if <0.05.*

Table 3 presents the univariate and multivariate logistic regression models predicting the likelihood of chronic pain in patients receiving OS. Based on the results of univariate logistic regression analyses, age (OR = 1.044; 95% CI: 1.003–1.087; *p* = 0.033) and early gabapentinoid medication after discharge from the hospital (OR = 3.526; 95% CI: 1.286–9.666; *p* = 0.014) predicted the outcome. However, in multivariate analyses, age was statistically non-significant (*p* = 0.077). Only early gabapentinoid medication predicted the outcome independently (aOR = 2.975; 95% CI: 1.055–8.388; *p* = 0.039).

**Table 3.** Logistic regression model predicting the likelihood of chronic pain in patients undergoing orthognathic surgery.

Variable		Univariate logistic regression analyses					Multivariable logistic regression analyses, default SEs				
		Coefficient	SE	OR	95% CI	*p*	Coefficient	SE	aOR	95% CI	*p*
Age, yr (continuous variable)		0.043	0.020	1.044	1.003–1.087	**0.033**	0.036	0.021	1.037	0.996–1.080	0.077
Sex (ref. male)		0.265	0.445	1.303	0.544–3.120	0.552					
Body mass index (continuous variable)		0.073	0.056	1.076	0.965–1.199	0.190					
Alcohol and/or drug abuse (ref. no)		1.006	0.811	2.734	0.558–13.409	0.215					
Combined oral contraceptive medication, women (ref. no)		−0.514	0.777	0.598	0.130–2.742	0.508					
Preceding mood disorder or neurotic, stress-related and somatofom disorder (ref. no)		−0.391	0.635	0.676	0.195–2.348	0.538					
Regular gabapentinoid medication after discharge from hospital (ref. no)		1.260	0.514	3.526	1.286–9.666	**0.014**	1.090	0.529	2.975	1.055–8.388	**0.039**
Dexamethasone (ref. low dose)		0.497	0.418	1.644	0.724–3.733	0.235					
Surgery type (ref. one jaw surgery)		−0.021	0.420	0.979	0.430–2.229	0.960					
Manipulation of the IAN (ref. no)		0.684	0.561	1.981	0.660–5.950	0.223					
Osteosynthesis type (ref. exclusively customized)											
	Standard	0.348	0.437	1.416	0.601–3.338	0.426					
	Combined	0.889	0.831	2.433	0.478–12.398	0.284					
Mandibular transfer (ref. set back)		1.011	1.039	2.748	0.359–21.045	0.330					

*CI: confidence interval; OR: odds ratio; SE: standard error; aOR: adjusted odds ratio; IAN: inferior alveolar nerve; ref.: reference category. The p-value is bolded if <0.05.*

## 5. Discussion

Contrary to our hypothesis, neither patient-specific nor surgical variables predicted chronic pain after OS. Higher age has previously been associated with prolonged neurosensory healing after nerve injury in OS [17, 21, 24, 25, 40]. In the present study, also an association, albeit not independent, was found. Even though in other surgical settings, preceding depression [27, 29, 34, 35, 36] and anxiety [27, 28, 29] have been associated with chronic postsurgical pain, no association was found here between preoperative psychiatric variables and outcome. However, our study only considered diagnosed disease states, *i.e.*, undiagnosed but symptomatic diseases were not surveyed. Interestingly, we found that patients with chronic postsurgical pain received gabapentinoids at discharge more often than patients without chronic postsurgical pain. We hypothesize that this reflects early postoperative pain burden or neuropathic features recognized by the treating clinicians. This result could indicate that OS professionals were able to identify patients at risk for chronic pain at discharge.

OS is an elective surgery and the occurrence of 7.9% of patients having chronic postsurgical pain at three months postoperatively warrants discussion. Although chronic pain tends to decrease over time (Figs. 1,2) and in most cases subsides within a year post-OS (Fig. 2), it affects a significant number of patients. Previous studies have reported rates of 0.5–10% for neuropathic pain after OS [3, 14, 41, 42, 43]. Postoperative pain conditions in OS have a negative effect on patients’ quality of life [44]. Considering that the purpose of OS is to increase patients’ oral health-related quality of life and correct functional limitations [45, 46, 47, 48] caused by the dentofacial deformity, the risk for chronic pain after OS should be thoroughly discussed with the patient.

Previously, some evidence has been proposed for the surgical technique being associated with possible neurosensory outcomes in OS. Compression or bending of the nerve during medial subperiosteal dissection [40], the direction and extent of mandibular movement [49], the type of internal fixation [50, 51] and choice of technique [52], and postoperative severe bleeding or swelling are associated with neurosensory disturbance outcomes. In this study, we did not find a correlation with the outcome when categorizing the movement of the mandible as advancement or setback or the type of osteofixation plate used for the osteosynthesis.

We found no correlation between the recorded type of injury to the nerve at the osteotomy site and chronic postsurgical pain (Table 3). Earlier studies have emphasized that injury to the IAN should be avoided during surgery; avoiding axonal nerve damage has been shown to prevent postsurgical pain in OS [43, 53]. Even if our findings can be explained by an undetected nerve injury or an inaccurate description of the severity of the nerve damage, assessing other aspects is important. A study by Jääskeläinen *et al*. [43] found that only 13% of patients with verified macroscopic or neurophysiological evidence of intraoperative axonal damage experience clinically significant neuropathic pain in OS a year after surgery. The type of injury to the nerve affects the healing and recovery from neurosensory symptoms. In general, demyelinating nerve injuries recover within four months after surgery [53], and regeneration has been shown six months [15] to a year post-OS [54]. Axonal injuries, however, show more incomplete sensory recovery than demyelinating injuries one year after surgery [43].

Based on prior research, age appears to be a promising variable for predicting postoperative chronic pain [3, 17, 55]. Our results also initially showed that chronic pain was more common in the group aged over 30 years, but in multivariate analyses age was non-significant. When comparing pain outcomes in other surgical settings, younger age appears to be a risk factor for chronic pain [56, 57]. Therefore, findings regarding age and chronic pain are somewhat contradictory.

Although we did not find an association between diagnosed presurgical psychiatric status and postoperative chronic pain, pain and psychological processes clearly influence one another. A biopsychosocial framework can be used to conceptualize pain; pain is a complex sensation that arises from the interrelationship of biological, social, and psychological factors [58]. Pain is affected by the individual’s previous pain experiences and is connected to the patients’ psychiatric history [27, 28, 29, 34] and psychological traits [8, 32, 33, 34]. In our study, patients with chronic postsurgical pain were more often prescribed early gabapentinoid medication (Table 3). This finding highlights the necessity of expanding the research to identify the factors that surgeons can recognize yet remain enigmatic in light of the results of the present study. Psychiatric status and psychosocial variables associated with chronification of pain should be evaluated at all stages of treatment and should be considered preoperatively, during postoperative healing and in patients dealing with chronic pain.

## 6. Limitations

The patients included in this study did not undergo a thorough psychiatric and psychosocial evaluation, which could mask the true prevalence of psychiatric illnesses in this population. A prospective study would provide a more accurate evaluation of the severity of injury to the IAN during surgery and other possible psychosocial variables not investigated in this study. Our regression model is also limited by the small number of outcome events, restricting the number of included variables. The independent association between early gabapentinoid medication and chronic pain should be interpreted with caution, as the wide confidence interval (95% CI: 1.055–8.388) reflects potential imprecision. Future studies with larger cohorts are needed to confirm these findings.

## 7. Conclusions

Our findings indicate that chronic postoperative pain is a potential drawback of OS, with an incidence of 7.9% at three months following surgery, declining to 1.6% at 12 months. No association between the surgical factors assessed and chronic postsurgical pain was detected in this study. Therefore, a more comprehensive understanding of such factors as psychosocial influences and individual resilience is necessary to identify patients at greatest risk of developing postoperative chronic pain.
